# Naringenin attenuates liver injury in *Schistosoma mansoni*-induced liver fibrosis and oxidative stress in mice model

**DOI:** 10.1371/journal.pntd.0013825

**Published:** 2025-12-19

**Authors:** Ting-Ruei Liang, Chao-Zong Liu, Shih-Yi Peng

**Affiliations:** 1 Ph.D. Program in Pharmacology and Toxicology, School of Medicine, Tzu Chi University, Hualien, Taiwan; 2 Department of Pharmacology, School of Medicine, Tzu Chi University, Hualien, Taiwan; 3 Department of Biochemistry, School of Medicine, Tzu Chi University, Hualien, Taiwan; Uniformed Services University: Uniformed Services University of the Health Sciences, UNITED STATES OF AMERICA

## Abstract

Schistosomiasis, a neglected tropical disease caused by parasitic trematodes of the genus *Schistosoma*, affects over 200 million individuals worldwide. Infection with *Schistosoma mansoni* remains a major public health challenge, leading to pathological conditions such as liver fibrosis, hepatosplenomegaly, and portal hypertension. The pathology of schistosomiasis is predominantly driven by the retention of parasite eggs within the liver, which induces granuloma formation and periportal fibrosis, culminating in significant hepatic injury. Granulomatous responses cause the infiltration of phagocytes and lymphocytes that secrete pro-inflammatory cytokines, subsequently activating hepatic stellate cells (HSCs). Activated HSCs promote excessive extracellular matrix deposition, driving fibrotic progression. Moreover, schistosomiasis-induced oxidative stress aggravates fibrosis by disrupting redox balance and enhancing HSC activation, leading to accelerating extracellular matrix deposition. Although praziquantel (PZQ) remains the standard treatment for schistosomiasis, its efficacy is limited to eliminating adult worms and does not extend to clear pre-existing eggs or directly resolve liver fibrosis. Therefore, adjunctive therapeutic strategies targeting fibrosis are needed. Naringenin, a flavonoid with potent hepatoprotective properties, has demonstrated anti-inflammatory and antifibrotic effects in various liver disease models. It exerts therapeutic effects by inhibiting HSC activation, attenuating collagen synthesis, and modulating profibrotic signaling pathways. Additionally, its antioxidant properties help mitigate oxidative stress, a key factor in fibrosis progression. This study utilizes a Balb/c mouse model of Schistosoma mansoni infection to evaluate the therapeutic potential of naringenin in reducing liver fibrosis, oxidative stress, and parasite burden.

## Author summary

Schistosomiasis is one of the most prevalent parasitic infections worldwide. *Schistosoma* eggs accumulate in the host liver, triggering inflammation and fibrosis. The pro-inflammatory cytokines induced by the eggs activate hepatic stellate cells (HSCs), which in turn produce large amounts of collagen, leading to liver fibrosis. If left untreated, liver fibrosis can progress to cirrhosis or even liver cancer. This study used a mouse model infected with *S. mansoni* to investigate the therapeutic potential of anti-fibrotic drugs. In vitro culture experiments revealed that naringenin could kill adult *Schistosoma* worms, damage their surface, and inhibit egg-laying by female worms. In vivo experiments showed that treating infected mice with naringenin for two weeks significantly reduced liver fibrosis. These findings highlight the potential of naringenin as a promising therapeutic agent for the clinical treatment of schistosomiasis and liver fibrosis.

## Introduction

Schistosomiasis, a parasitic disease caused by Schistosoma infection, remains a major public health problem worldwide in the 21^st^ century and is recognized by the World Health Organization (WHO) as one of the most significant tropical diseases, alongside malaria. According to WHO statistics, approximately 254 million people were infected globally in 2021, with 105.4 million requiring treatment. The disease causes around 15,000 deaths annually, and nearly 600 million people in developing countries are at risk of schistosomiasis [1]. The three main *Schistosoma* species that infect humans are *S. mansoni*, *S. japonicum*, and *S. haematobium*. Clinical symptoms include hepatosplenomegaly, liver fibrosis, portal hypertension, and cirrhosis, which can ultimately result in death.

The severity of schistosomiasis symptoms is primarily associated with the intensity of cercarial infection, with the eggs serving as the main pathological agents in the definitive host. When cercariae penetrate the skin of definitive host, they circulate through the bloodstream, develop, and mate in the liver. Subsequently, they migrate to the mesenteric venous plexus and lay eggs [2].

The eggs travel through the portal vein to the liver, where they primarily accumulate. These eggs secrete soluble egg antigens (SEAs), which stimulate T cells to release cytokines, thereby triggering an inflammatory response. This immune activation leads to granuloma formation around the eggs, involving T cells, neutrophils, eosinophils, fibroblasts, and other immune cells. [3] Over time, these granulomas progress to liver fibrosis. Liver fibrosis, caused by chronic liver damage and inflammation, lead to the excessive deposition of extracellular matrix components (ECM), including collagen, elastin, and fibronectin, in the liver [4]. In response, host immune cells produce various pro-inflammatory mediators, including IL-1β, IL-18, TNF-α, and T helper 2 (Th2) cytokines (e.g., IL-4 and IL-5), thereby triggering an intense immune response in the infected host [5–7]. Currently, effective anti-fibrotic therapies remain limited. Although the widespread use of the antiparasitic drug praziquantel has proven beneficial, it cannot fully prevent the progression of chronic schistosomiasis to advanced stage or the development of liver fibrosis in affected patients.

In recent years, the search for new drugs from phytochemicals and animal-derived toxins has become a growing trend. Flavonoids, low-molecular-weight phenolic compounds, are abundantly distributed across various plant species. They are commonly found in herbs, fruits, stems, vegetables, nuts, cereals, seeds, and flowers [8–10]. They serve as signaling molecules, allelopathic compounds, phytoalexins, antidotes, and antimicrobial defense compounds [11,12].

Flavonoids exhibit antioxidant [13], anti-inflammatory [14], antimutagenic, anticancer [15,16], antimicrobial [17], anti-fibrotic properties [18]. Their ability to modulate key cellular enzyme functions further highlights their significance to human health [8]. In the context of liver diseases, flavonoids have been shown to attenuate pro-inflammatory cytokine release by inhibiting NF-κB activation. They also increase adiponectin levels, enhance glucose tolerance and insulin sensitivity, improve dyslipidemia, and lower blood pressure in patients with non-alcoholic fatty liver disease (NAFLD) [19]. In addition, several flavonoids have demonstrated anti-fibrotic effect by suppressing activation of hepatic stellate cells and reducing deposition of extracellular matrix (ECM) in fibrotic tissue [18]. Naringenin, a flavanone-class flavonoid compound predominantly found in citrus fruits, has drawn considerable attention due to its multiple biological activities, including anti-inflammatory, antioxidant, and hepatoprotective effects [20,21]. It has shown therapeutic potential in various fibrotic diseases by modulating the TGF-β1 pathway and other related signaling cascades [22]. Additionally, it exhibits a strong capacity to scavenge reactive oxygen species (ROS), thereby alleviating oxidative stress [23].

This study aims to evaluate the efficacy of naringenin against *Schistosoma mansoni* adult worms in vitro, as well as its therapeutic effects in mice infected with *S. mansoni*, focusing on its anti-fibrotic and anti-inflammatory properties. Furthermore, adult worm cultures treated with naringenin will be observed to assess parasite viability and morphological changes, thereby exploring the potential of naringenin as a therapeutic agent for schistosomiasis-induced liver fibrosis.

## Materials and methods

### Ethics statement

All animal protocols received approval from the Institutional Animal Care and Use Committee (IACUC) of Tzu Chi University, Taiwan (Approval No. 111069). Experimental protocols were conducted in compliance with the guidelines of the National Institutes of Health (NIH) Guide for the Care the Use of Laboratory Animals (DHHS Publication No. NIH85–23, revised 1996)

### Parasite and animals

Our laboratory maintains a *Schistosoma mansoni* (Puerto Rican strain) life cycle, originally obtained through the Biomedical Research Institute (Rockville, MD, USA; ZIP: 20852). *Biomphalaria glabrata*, a freshwater snail, was employed in the role of the intermediate host, with male BALB/c mice acting as definitive host. BALB/c mice were purchased from the National Laboratory Animal Center in Taipei. Animals were kept in a controlled environment at 25 ± 2°C under a 12-hour light/dark cycle, with unrestricted access to food and water.

### Naringenin

**Fig 1:**
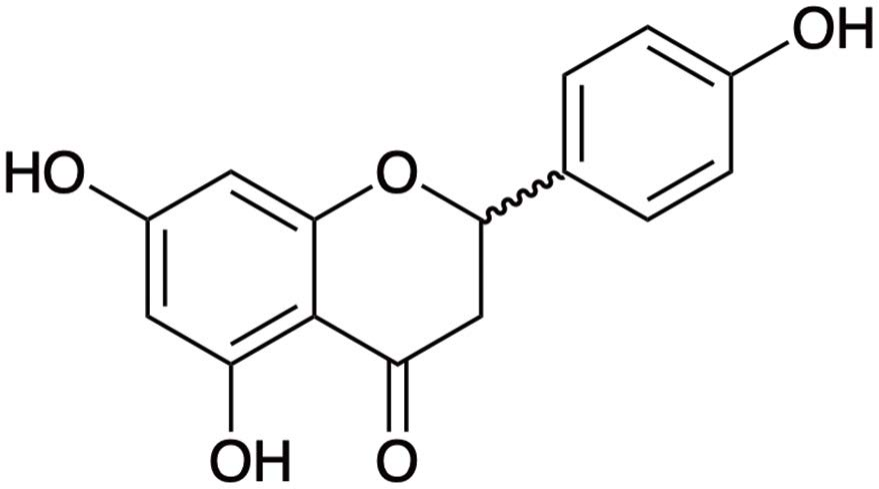
Chemical structure of naringenin. The chemical structure of naringenin (C_15_H_12_O_5_; molecular weight 272.25 g/mol), the flavanone compound used for both in vitro and in vivo experiments in this study.

Naringenin (C_15_H_12_O_5_, molecular weight 272.25 g/mol), a flavanone naturally found in citrus fruits, was purchased from Sigma-Aldrich Co. (N5893, Sigma, St.Louis, MO, USA). The chemical structure of naringenin is shown in Fig 1. For the in vitro experiments, naringenin was dissolved in DMSO (Sigma, St. Louis, MO, USA) and the concentration was adjusted to 100 μg/ml and 200 μg/ml, respectively. For the in vivo experiments, mice were initially administered 50 mg/kg/day of naringenin suspended in distilled water via oral gavage using a stainless steel feeding tube.

### In vitro culture experiments

The purpose of this experiment was to test the ability of *naringenin* to destroy *S. mansoni* adult worms and inhibit female egg-laying in vitro. *S. mansoni* adult worms, obtained by the venous perfusion method [1], were maintained in DMEM culture medium (Gibco; Thermo Fisher Scientific, Inc.) containing 10% FBS and 1% penicillin-streptomycin. The worms were cultured in 1 mL of medium per well in a 24-well plate. The worms were initially maintained in culture medium for 24 hours. Afterward, they were treated with either 100 μg/mL or 200 μg/mL naringenin. Praziquantel at 50 μg/mL was applied as a positive control, while culture medium alone served as a negative control. Plates were incubated in a cell culture incubator set to 37℃ and 5% CO_2_. The morphology and survival rate of the worms were monitored daily using a dissecting microscope for one week. Worm survival rates were classified as follows: 0 - dead; 1 - motionless but responsive to acupuncture stimulation; 2 - fully active [7]. Additionally, to study the effect of flavonoids on the reproductive capacity of adult worms, the medium was collected and renewed daily, and egg production was recorded. After 7 days of culture, worms were harvested, thoroughly washed with PBS, fixed in 2.5% glutaraldehyde at 4°C for one hour, and subsequently rinsed with 5% sucrose. Afterward, they were incubated in 1% osmium tetroxide for one hour, dehydrated through a graded ethanol series (50%-100%, 10 minutes each), critically point-dried, and gold-coated. Imaging was performed using the HIRACHI S-4700 field emission scanning electron microscope (Hitachi Ltd, Tokyo, Japan).

### Animal infection and treatment

Forty-five six-week-old male BALB/c mice were randomly and evenly assigned to three groups: an uninfected control group, an infection group (10 weeks post-infection), and a treatment group (8 weeks post-infection followed by treatment with 50 mg/kg/day naringenin for two weeks) [24] (Fig 3A). The infection and treatment groups of mice were infected percutaneously through the tail with 100 ± 10 cercariae, while the control group received distilled water as a placebo. All animals were euthanized at ten-week post-infection. From each group, five mice were used to assess worm burden, while the others were sacrificed for sample collection to perform biochemical analysis, histological examination, and western blot analysis.

**Fig 2:**
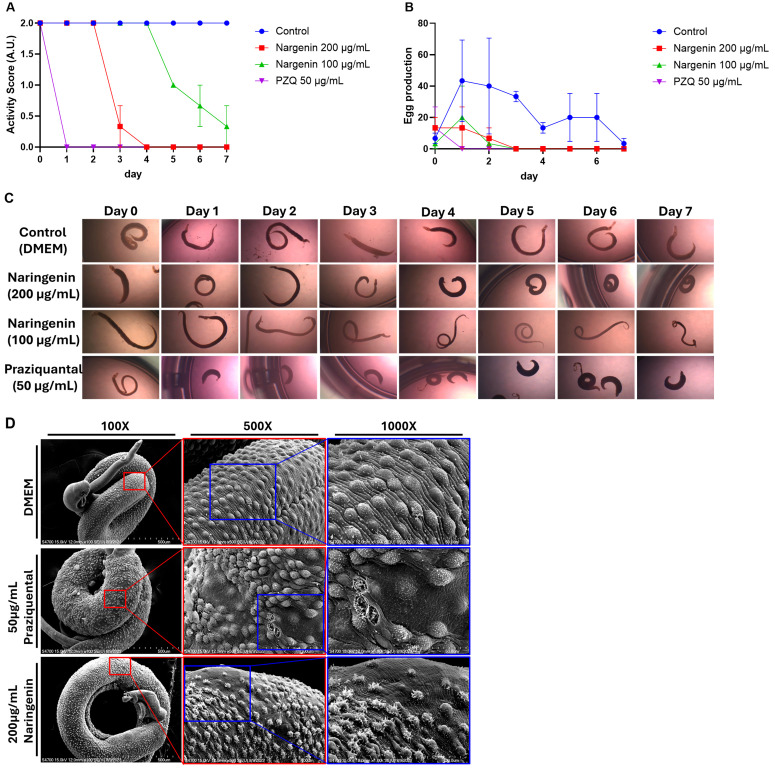
Naringenin reduces adult worm motility and egg laying, and induces tegumental damage in schistosomes in vitro. **(A)** Activity score of adult worm. On the fourth day of continuous treatment with 200 μg/mL naringenin, the activity of adult worms was significantly reduced. **(B)** Number of eggs collected from the culture medium daily. **(C)** Representative images of adult worms cultured treatment in vitro with different treatments, observed daily. **(D)** Scanning electron microscopy images showing the surface morphology of adult worms collected after 7 days of in vitro culture.

### Adult worm burden

*Schistosoma mansoni* adult worms are primarily located in the inferior mesenteric vein and the liver. After anesthetizing the mice, perfusion fluid was pumped into the descending aorta. The perfusion fluid passed through the mesenteric vein and liver, then exited through the liver portal vein and was collected in a culture dish. The worms were then identified and counted using a dissecting microscope. The collected worms were washed several times with PBS and subsequently prepare for scanning electron microscopy (SEM) analysis as previously described.

### Schistosome eggs count

Schistosome eggs were counted in liver and large intestinal tissues. Approximately 0.2 g of liver and large intestinal tissues were collected and incubated with 4% KOH at 37°C for 4 hours. After incubation, the mixture was centrifuged at 1500 rpm for 15 minutes, after which the supernatant was discarded. The collected pellet was resuspended in 500 µl of phosphate-buffered saline (PBS). Then, 100 µl of the suspension from each sample was taken and counted under a microscope.

### Serum biochemical analysis

Whole blood was harvested via cardiac puncture, allowed to clot at room temperature for 20 minutes, and then centrifuged at 800 xg for 20 minutes to collect serum. The serum was stored at −80°C until further analysis. Serum levels of aspartate transaminase (AST) and alanine transaminase (ALT) were subsequently measured using GOT (ASAT) and GPT (ALAT) IFCC mod liquiUV reagents (LOT 12021, 12022; HUMAN GmbH, Wiesbaden, Germany).

### Histological tissue preparation and staining

Liver tissues from each group were collected and promptly fixed in 10% neutral buffered formalin for 24–48 hours at room temperature. Fixed tissues were dehydrated in ascending ethanol series (70% to 100%), with each step lasting 1 hour, and cleared in Sub-X xylene substitute. Subsequently, tissues were embedded in paraffin to form tissue blocks. Sections were cut using a microtome and mounted on glass slides. Prior to staining, the sections were incubated at 65℃ for 30 minutes, deparaffinized in Sub-X, and rehydrated via descending ethanol concentrations (100% twice, 95%, 85%, 75%, and 50%) followed by rinsing in distilled water. Histological staining was performed on rehydrated sections following standard protocols as described in a previously study [7]. Hematoxylin and eosin (H&E) staining was used to evaluate liver injury, while Masson’s trichrome staining (Trichrome Stain Kit, Modified Masson’s; ScyTek Laboratories, Cat. No. TRM-1) and Picro-Sirius Red staining (Picro-Sirius Red Stain Kit [For Collagen]; ScyTek Laboratories) were performed to assess collagen deposition and fibrosis.

### Histopathological examination

For H&E staining, hepatic injury was evaluated based on the following scoring criteria. A score of 0 indicated no evidence of injury; 1 represented mild damage with cytoplasmic vacuolation and focal nuclear pyknosis; 2 indicated moderate to severe injuries, characterized by extensive pyknosis and loss of intercellular borders; 3 denoted severe necrosis with hepatic cords disintegration, congestion, and neutrophil infiltration [25]. In Masson’s trichrome staining and Picro-Sirius Red staining, fibrotic regions were specifically stained blue and red, respectively. Fibrotic regions were identified based on specific staining patterns, manually delineated, and their relative areas quantified using ImageJ. (Version 1.46, National Institute of Health, Bethesda, MD, USA).

### Protein extraction

The total protein from the liver tissue of each experimental group of mice was extracted to detect changes in expression of fibrosis- and inflammation-related proteins. Briefly, liver tissues were mixed with 800 μL RIPA lysis buffer and homogenized using a glass homogenizer. The tissue lysates were then centrifuged at 12,000 × g for 15 minutes at 4°C, and the supernatants were filtered through 0.45 μm syringe filters to collect soluble proteins. The protein concentration was determined using the Bio-Rad Protein Assay Kit (Bio-Rad Laboratories Inc, California, USA).

### Western blotting

SDS-PAGE was conducted to separate sample proteins from each experimental group, followed by transfer onto a 0.45μm PVDF membrane (Millipore) using the Mini Trans-Blot Electrophoretic Transfer Cell (Bio-Rad). The membrane was blocked with 5% non-fat milk for 1 hour and washed three times with PBS-T (0.05% Tween 20 in PBS). Subsequently, the membrane was incubated overnight at 4℃ with primary antibodies: α-tubulin(A6830; ABclonal, USA), TGF-β (A2124; ABclonal, USA), α-SMA (A7248; ABclonal, USA), collagen I (A5786; ABclonal, USA), fibronectin (A12932; ABclonal, USA), Smad4 (A5657; ABclonal, USA), Smad7 (A12343; ABclonal, USA), IL-18 (A1115; ABclonal, USA), IL-1β (A1112; ABclonal, USA), iNOS (A3774; ABclonal, USA), SOD2 (A1340; ABclonal, USA), and GSR (A4566; ABclonal, USA) which diluted 1:1000 in antibody dilution buffer (sterile PBS with 0.05% ProClin300). Following incubation with the primary antibodies, the membrane was washed three times with PBS-T and incubated with horseradish peroxidase (HRP)-conjugated secondary antibodies (Goat Anti-Mouse IgG Antibody, Peroxidase Conjugated, H + L, AP124P, Millipore; Goat Anti-Rabbit IgG Antibody, HRP-conjugate, AP187P, Millipore) diluted 1:10000 for 1 hour at 4°C. Following another three washes with PBS-T, the membrane was treated with Immobilon Western Chemiluminescent HRP substrate (Millipore) and visualized using chemiluminescence detection. Targeted protein levels were normalized to α-tubulin as an internal control, and quantification was performed using ImageJ software.

### Statistical analysis

All experimental data were analyzed and visualized using GraphPad Prism 9.5 software (GraphPad Software Inc., San Diego, CA, USA). Data are presented as the mean ± standard deviation. One-way ANOVA with Tukey’s post hoc test was performed to assess differences among groups. A p-value < 0.05 was considered statistically significant and indicated by asterisks.

## Results

### Naringenin inhibits adult worm activity and egg laying, and induces surface damage to schistosomes in vitro

In the PZQ-treated group (positive control), one day after treatment, adult worms exhibited whole-body contraction, curling, and losing their ability to extend. The activity score of adult worms was reduced to zero. Male worms had completely lost motility, while a few female worms, residing within the male gynecophoral canal, still displayed slight twitching. In the 200 μg/mL naringenin-treated group, the activity score markedly decreased by day 3 and was completely lost by day 4. In comparison, the 100 μg/mL group exhibited a delayed response, with worm activity beginning to decline on day 5 and progressively decreasing thereafter. Additionally, it was observed that worms in the naringenin-treated group did not exhibit whole-body contraction, seen in PZQ-treated group (Fig 2A and 2C). Regarding egg-laying ability, no egg production was detected in PZQ-treated group, as the worms had completely lost motility. In all naringenin-treated groups, regardless of concentration, egg production was reduced compared to the control group and ceased entirely by day 3 (Fig 2B). Scanning electron microscopy (SEM) was used to evaluate surface damage in *Schistosoma* worms. Both naringenin and praziquantel (PZQ) induced damage to the surface tubercles of *Schistosoma* worms. In the PZQ-treated group, the number of tubercles was reduced and many appeared disrupted. In contrast, worms in the naringenin-treated group exhibited tubercles with a more spiked or irregular morphology. The tegument of worms in the control group (DMEM) remained relatively intact, with well-preserved surface structures (Fig 2D).

### Naringenin ameliorates *Schistosoma mansoni*-induced hepatic fibrosis by improving liver pathology and reducing collagen deposition in a Balb/c mouse model

**Fig 3:**
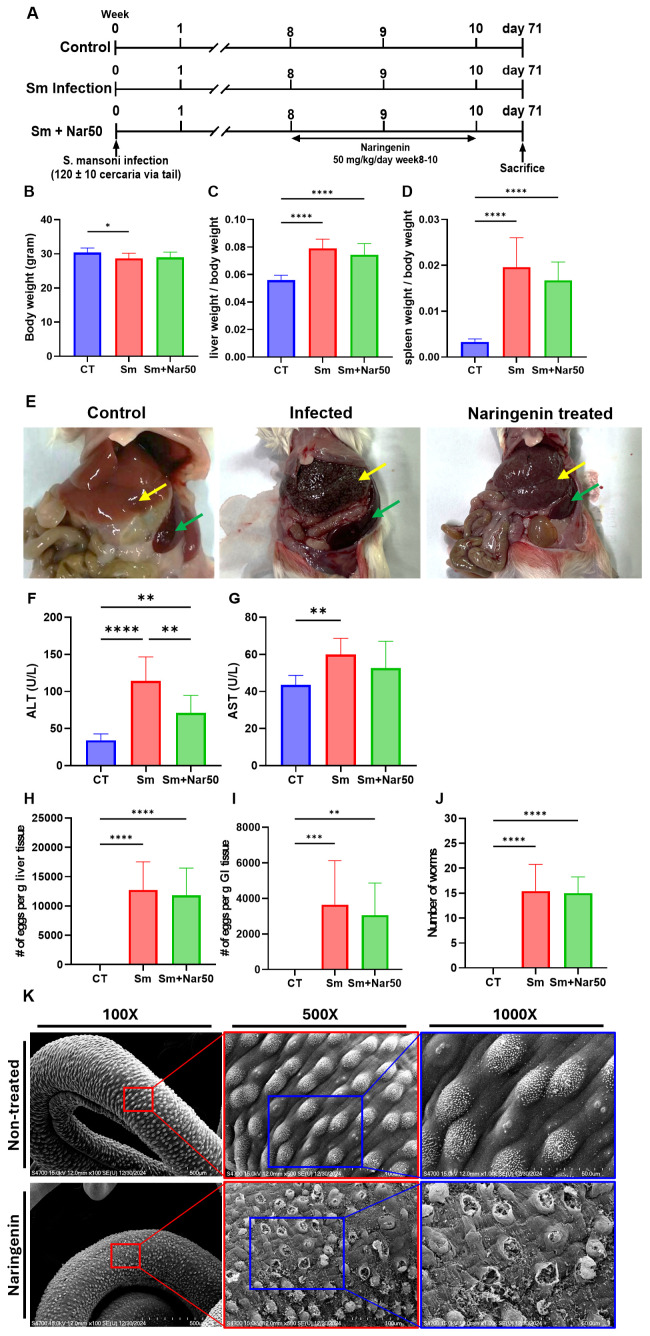
Naringenin alleviates liver injury and pathology in S. mansoni-infected Balb/c mice. (A) Experimental design and workflow. (B) Body weights. (C) Liver weight index (the ratio of liver weight to body weight). (D) Spleen weight index (the ratio of spleen weight to body weight). (E) Representative images of gross liver and spleen morphology from control mice, S. mansoni-infected mice, and naringenin-treated infected mice. (liver, yellow arrows; spleen, green arrows) Notably, visible lesions and hepatosplenomegaly were observed in the livers and spleens of infected mice. Serum level of (F) ALT and (G) AST were measured to assess liver function. Number of eggs collected per gram of mouse (H) liver and (I) gastrointestinal (GI) tract. (J) Worm burden recovered from mice by perfusion. Data are expressed as mean ± SD. Statistical significance was assessed relative to the control or infection group: *P < 0.05, **P < 0.01, ***P < 0.001, ****P < 0.0001. (K) Representative SEM images showing the surface morphology of adult worms recovered from mice by perfusion.

Mice were sacrificed for the measurement of body, liver, and spleen weights. The liver weight index and spleen weight index were calculated by dividing the liver and spleen weights by the total body weight of each mouse, respectively. The naringenin-treated group exhibited a slight increase in body weight, accompanied by a mild reduction in liver and spleen weight indices relative to the infection group, suggesting moderate improvements in hepatosplenomegaly (Fig 3B-D). Furthermore, the number of *Schistosoma mansoni* eggs in the liver and intestines was quantified, with no significant differences observed between the infection and treatment groups (Fig 3H-I). Similarly, the number of adult worms perfused from mice showed the same trend (Fig 3J). Scanning electron microscopy (SEM) was also used to observe surface damage in *Schistosoma* worms, revealing morphological alterations similar to those observed in the in vitro experiments (Fig 3K).

**Fig 4:**
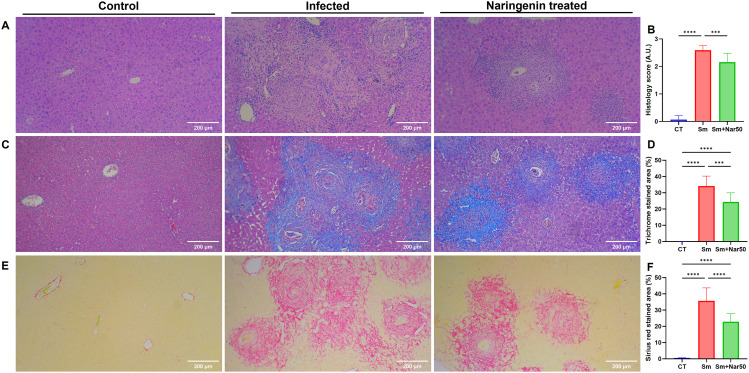
Naringenin treatment attenuates liver fibrosis in S. mansoni-infected mice. **(A)** Hematoxylin and eosin (H&E) staining of mouse liver tissue sections and **(B)** quantification of histology score. **(C)** Masson’s Trichrome staining of mouse liver tissue sections. **(D)** Quantification of trichrome-stained areas. **(E)** Picro-Sirius Red staining of mouse liver tissue sections. **(F)** Quantification of Sirius red-stained areas. For each group, quantification was performed on 10 slides with ten random microscope fields per slide. Result data are expressed as mean ± SD (n=10). Statistical significance was assessed relative to the control or infection group: *P < 0.05, **P < 0.01, ***P < 0.001, ****P < 0.0001.

The liver and spleen lesions were compared among the three experimental groups. In the infected group, the liver and spleen were significantly enlarged, with numerous white spots on the liver caused by the accumulation of eggs, and the liver color changed from normal red to dark brown. In contrast, the treatment group exhibited milder liver lesions and a brighter liver color compared to the infected group (Fig 3E). Liver injury and fibrosis in each experimental group were assessed using H&E, Masson’s trichrome, and Picro-Sirius Red staining. According to the H&E staining result, the infection group exhibited granuloma formation, infiltration of neutrophils and eosinophils, severe hemorrhage, and necrosis. In contrast, these pathological features were markedly reduced in the treatment group. The liver injury index quantitatively confirmed a significant reduction in the treatment group (Fig 4A-B). Masson’s trichrome and Picro-Sirius Red staining were conducted to evaluate collagen deposition and liver fibrosis. In Masson’s trichrome staining, collagen is stained blue, whereas in Picro-Sirius Red staining, it appears red. Fibrotic areas were manually selected using ImageJ, and the corresponding regions were quantified to determine the extent of fibrosis. Fibrotic area was markedly decreased in the treatment group relative to the infection group. Liver function markers showed consistent results (Fig 4C–4F). Serum AST and ALT levels were significantly elevated in the infection group but markedly reduced following treatment, indicating an improvement in liver function (Fig 3F–3G). In conclusion, naringenin effectively improved liver pathology and function in *Schistosoma mansoni*-infected mice.

### Naringenin attenuates liver fibrosis through downregulation of fibrosis-related proteins expression

**Fig 5:**
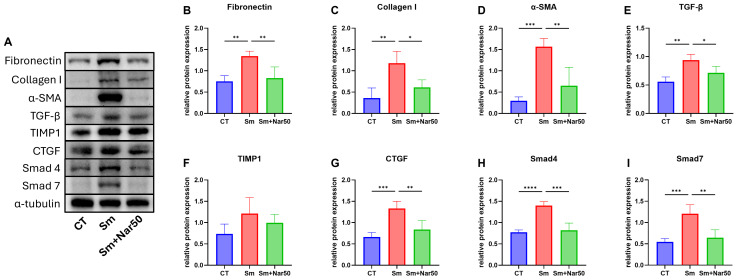
Naringenin regulates the expression of fibrotic proteins in mouse livers. **(A)** Representative western blot images showing fibrosis-related protein level. **(B-I)** Protein expression levels of fibrotic marker, including Fibronectin, Collagen I, α-SMA, TGF-β, TIMP-1, CTGF, Smad4, and Smad7, normalized to α-tubulin. Data are presented as mean ± SD. (n=4) Statistical significance was determined by comparison to the control group or the infection group: *P < 0.05, **P < 0.01, ***P < 0.001, ****P < 0.0001.

To further investigate the therapeutic effect of naringenin on liver fibrosis induced by *S. mansoni*, this study examined the expression of fibrosis-related proteins by western blotting. One of the key events in liver fibrosis is the activation of hepatic stellate cells (HSCs). Therefore, the target proteins analyzed included α-SMA, TGF-β, collagen I, fibronectin, CTGF, TIMP1, Smad4, Smad7 (Fig 5A–5I). Protein expression levels were normalized to α-tubulin, which served as an internal control. Our results showed that all target proteins were elevated in the infection group and reduced in the naringenin-treated group.

### Naringenin regulates the expression of oxidative stress- and inflammation-related proteins

**Fig 6:**
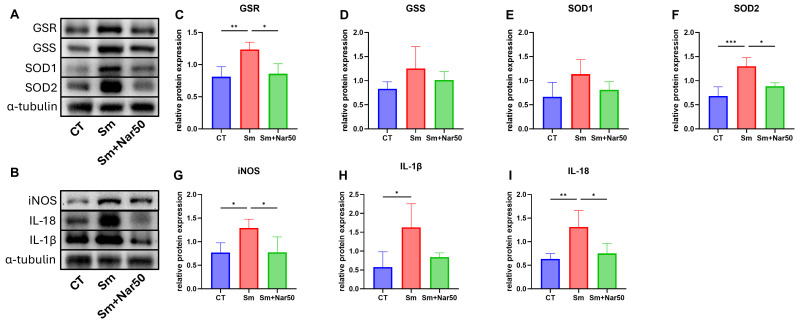
Naringenin modulates the expression of oxidative stress-related and inflammatory proteins in mouse livers. **(A, B)** Representative western blot images showing the expression levels of inflammatory and oxidative stress-related markers. **(C–F)** Protein expression levels of oxidative stress-related proteins, including GSR, GSS, SOD1, and SOD2, normalized to α-tubulin. **(G–I)** Protein expression levels of inflammatory proteins, including iNOS, IL-1β, and IL-18, also normalized to α-tubulin. Data are presented as mean ± SD. (n=4) Statistical significance was determined relative to the control or experimental group: *P < 0.05, **P < 0.01, ***P < 0.001, ****P < 0.0001.

In addition to its anti-fibrotic effects, we explored whether naringenin also modulates oxidative stress and inflammation in *S. mansoni*-induced liver pathology. In the context of oxidative stress, GSS and GSR catalyze synthesis and reduction of glutathione. SOD1 and SOD2 are involved in degradation of superoxide radicals. iNOS, which promotes the formation of reactive nitrogen species (RNS), is also implicated in the amplification of inflammatory response. Pro-inflammatory cytokines, including IL-1β and IL-18, further promote hepatic inflammation. Western blot analysis showed that naringenin treatment resulted in a decreased level of oxidative stress- and inflammation-related proteins compared to the infection group (Fig 6A–6I).

## Discussion

Schistosomiasis affects more than 200 million people globally each year, with liver fibrosis and hepatosplenomegaly being the primary clinical manifestations [26,27]. Although praziquantel is effective in eliminating adult schistosomes, it does not remove the deposited eggs within host tissues, nor does it reverse the established organ fibrosis. In recent years, numerous studies have investigated the biological activities of phytochemicals and plant secondary metabolites, particularly flavonoids. Naringenin, a flavonoid extracted from citrus fruits, has been extensively investigated for its antioxidant and anti-inflammatory properties, which contribute to its therapeutic potential in various diseases [28].

In our study, the effects of naringenin on *Schistosoma mansoni* adult worms were first examined in vitro. Naringenin was found to reduce the motility of the adult worms and led to their death. Previous studies have proposed several mechanisms by which small-molecule compounds affect adult Schistosoma worms. Among them, the most commonly reported effect is disruption of the worm’s tegument, a critical outer structure responsible for evading the host immune response. Some natural compounds, such as licochalcone A, have been shown to induce mitochondrial swelling, degradation of mitochondria, and chromatin condensation in the nucleus, leading to excessive free radical production in adult worms. Other compounds have been reported to inhibit *S. mansoni* ATPase and ADPase activities, which may impair the energy production [29]. In contrast, praziquantel (PZQ), the commonly used anti-schistosomal drug, appears to act through a different mechanism. Although its exact mode of action remains unclear, some studies suggest that PZQ interferes with ion channel activity, thereby increasing calcium ion permeability, which ultimately results in worm tetanic contraction and death. In our study, adult worms treated with praziquantel (PZQ), used as the positive control, exhibited marked contraction. In contrast, naringenin-treated worms did not show noticeable body contraction but instead exhibited disruption of the tegument.

In vitro culture with naringenin showed that a reduction in the number of surface tubercles on male *Schistosoma mansoni* was observed, compared to those maintained in DMEM alone (negative control), in which the tegumental structures appeared more intact. This observation was further supported by scanning electron microscopy (SEM) analysis.

Moreover, in both the PZQ- and naringenin-treated groups, female worms that remained pair with males appeared less affected. In some cases, even when the male worm had completely lost motility, the female worm within the gynecophoral canal still exhibited slight movement. Naringenin was also found to suppress the egg-laying capacity of *Schistosoma mansoni*. Notably, egg production was reduced even when the worms still exhibited motility, suggesting that naringenin may impair reproductive function before causing overt effects on worm viability.

Although naringenin reduced egg production and induced mortality of worms in vitro, the effects were not observed *in vivo*, where egg retention in organs, including liver and intestines, as well as adult worm burden, remained unchanged. However, naringenin treatment can also cause surface damage to adult worms in vivo, which suggests that it remains biologically active in vivo.

Moreover, naringenin-treated mice showed improved liver function markers compared to the infected group, suggesting that it may alleviate hepatic injury despite continued egg deposition. Histological analysis using H&E staining of liver sections further confirmed that naringenin significantly ameliorated tissue damage.

The primarily pathological damage caused by *Schistosoma* infection is from the host immune response to egg deposition, which leads to immune dysregulation, chronic inflammation, and ultimately tissue fibrosis. To evaluate hepatic fibrosis, Masson’s trichrome and Picro-Sirius Red staining were applied to liver sections to visualize and quantify collagen deposition. Treatment with naringenin significantly reduced the fibrotic area, indicating its potential anti-fibrotic effects.

Liver fibrosis is caused by microbial (e.g., parasitic or viral) infections or metabolic chronic liver diseases poses a major global health challenge. Liver fibrosis is primarily driven by the activation of hepatic stellate cells (HSCs) in response to external stimuli. Upon activation, HSCs transdifferentiate into collagen-producing myofibroblasts. This process is originally part of a physiological repair mechanism designed to resolve short-term injury. Under normal conditions, it is tightly regulated by anti-fibrogenic mechanisms, such as matrix metalloproteinases (MMPs) and tissue inhibitors of metalloproteinases (TIMPs), to prevent excessive fibrotic responses [30, 31]. However, under conditions of chronic inflammation, the sustained activation of hepatic stellate cells (HSCs) disrupts the balance of extracellular matrix (ECM) deposition, thereby promoting the progression of liver fibrosis [32].To investigate the anti-fibrotic mechanism of naringenin, we conducted western blot analysis to examine fibrosis-related markers. Upon activation, HSCs upregulate the expression of α-smooth muscle actin (α-SMA), collagen I, and fibronectin [33].Our results showed that naringenin markedly reduced the expression of these proteins. In addition, we observed that both transforming growth factor-beta (TGF-β), a major upstream activator of HSCs, and connective tissue growth factor (CTGF), a downstream mediator of fibrogenic responses, were downregulated following naringenin treatment. As previously mentioned, the anti-fibrogenic response involves matrix metalloproteinases (MMPs), which degrade collagen. During chronic inflammation, however, cells produce high levels of tissue inhibitors of metalloproteinases (TIMPs), particularly TIMP1, which inhibit MMP activity and block collagen degradation. Notably, naringenin treatment slightly decreased TIMP1 expression, which may partially contribute to the restoration of ECM balance. Previous studies have demonstrated that Smad4 facilitates the activation of the fibrosis signaling pathway, whereas Smad7 acts as an inhibitory regulator. During hepatic stellate cell (HSC) activation, the expression of Smad4 typically increases, while that of Smad7 decreases [34]. In our study, both Smad4 and Smad7 protein levels were increased following *Schistosoma* infection and were reduced after naringenin treatment, as shown by western blot analysis.

Naringenin are known for their anti-inflammatory and antioxidant properties. Given that chronic inflammation and oxidative stress are key contributors to schistosomiasis-induced pathology, we further investigated whether naringenin exerts similar effects in *Schistosoma mansoni*-infected mice. Previous studies have shown that schistosome eggs accumulated in the liver stimulate macrophages to release inducible nitric oxide synthase (iNOS). While iNOS plays a physiological role by producing nitric oxide to generate oxidative stress that suppresses parasite development and modulates the Th1/Th2 balance [35], excessive and sustained iNOS expression may lead to chronic inflammation. In our study, naringenin treatment effectively suppressed iNOS expression. Moreover, schistosome eggs can activate the NLRP3 inflammasome, which lead to the release of IL-1β and IL-18 [36]. Consistently, our results demonstrated that naringenin decreased IL-1β and IL-18 expression.

Regarding oxidative stress, various phagocytic cells such as eosinophils and neutrophils produce high levels of reactive oxygen species (ROS) as a defense mechanism against *Schistosoma*. In response, the host activates antioxidant defense systems, including the use of superoxide dismutase (SOD) to degrade superoxide anions (•O_2_^−^), the synthesis of glutathione via glutathione synthetase (GSS), and the reduction of oxidized glutathione through glutathione reductase (GSR). Naringenin have been reported to neutralize ROS [37]. In our study, naringenin treatment led to a reduction in the expression of antioxidant proteins compared to the *S. mansoni*-infected group. This suggests that naringenin may relieve oxidative stress and reduce the burden on the host’s antioxidant defense system.

Overall, naringenin demonstrates tegumental damage to adult worms in vitro and exhibits therapeutic potential in attenuating *Schistosoma*-induced liver injury through its anti-fibrotic, anti-inflammatory, and antioxidant properties.

Similarly, various flavonoid compounds have been reported to exert therapeutic potential in schistosomiasis. For example, rutin and litchi seed extracts have been shown to alleviate schistosomiasis-related pathology [38,39]. Robustic acid and alpinumisoflavones from *Millettia thonningii* seeds demonstrated biological activity against *S. mansoni* in vitro [40], while flavonoids derived from Styrax plants exhibited adult worm-killing effects [41]. Besides, isoflavones, such as curcumin, genistein, quercetin, and silymarin have shown antiparasitic potential against various trematodes, likely through disruption of the tegument and modulation of parasite metabolism [42]. These studies support the notion that flavonoid compounds represent a promising class of candidates for the treatment of schistosomiasis.

A limitation of the current study is that although naringenin effectively alleviates liver injury and fibrosis, its ability to damage adult worms *in vivo* remains limited. Consequently, further investigation is warranted to evaluate the potential therapeutic benefits of combining naringenin with praziquantel (PZQ). In the future, co-administration of PZQ and naringenin may not only enhance the elimination of adult worms but also promote the reversal of liver fibrosis caused by *Schistosoma mansoni* infection. This combinatory approach may offer an effective strategy for the clinical management of schistosomiasis and its associated hepatic complications.

## Supporting information

S1 Dataset All relevant dataset. (XLSX)

S1 Fig Original blots for Fig 5A. (TIF)

S2 Fig Original blots for Fig 6A and B. (TIF)
