# Supplementary figures and images for: Naringenin attenuates liver injury in Schistosoma mansoni-induced liver fibrosis and oxidative stress in mice model
Source: PLoS Negl Trop Dis. 2025 Dec 19;19(12):e0013825. doi: 10.1371/journal.pntd.0013825 (PMC12716769; doi:10.1371/journal.pntd.0013825)

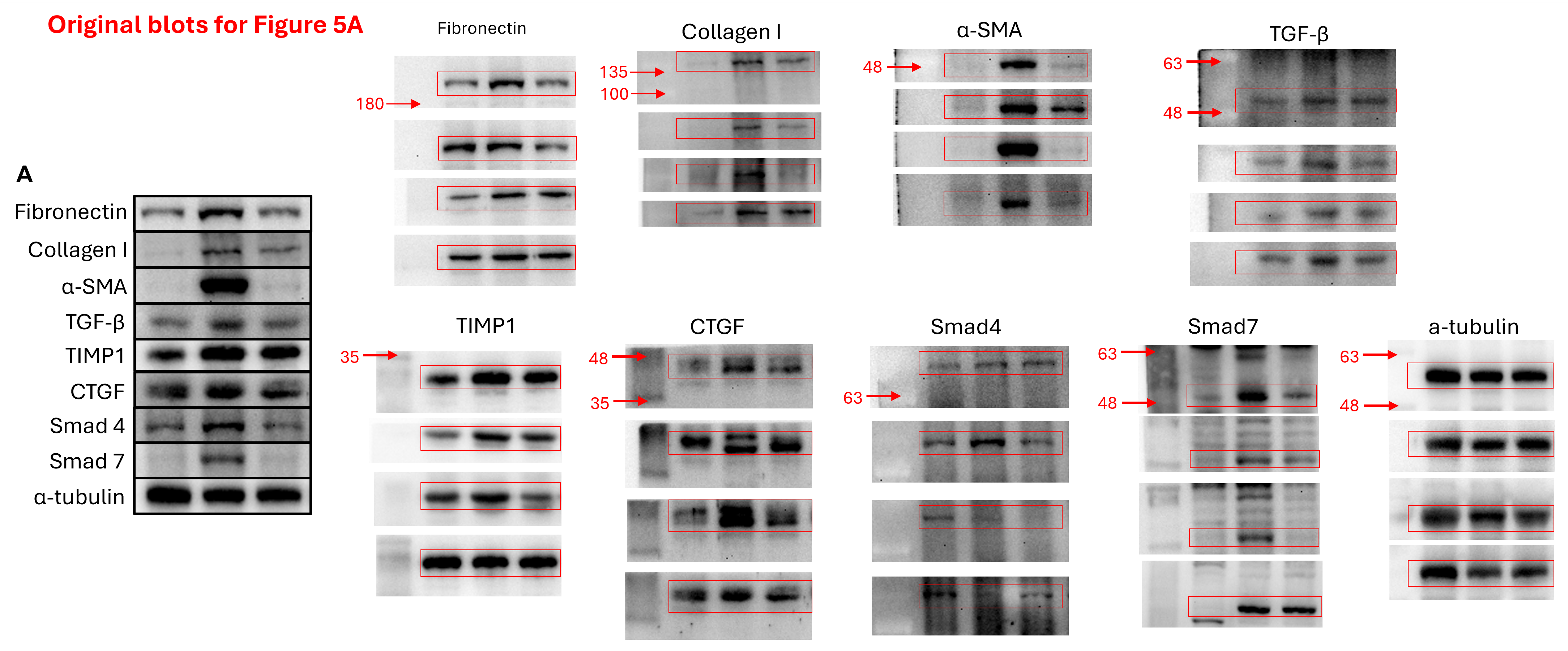

Supplement: S1 Fig — (TIF) [file pntd.0013825.s002.tif]

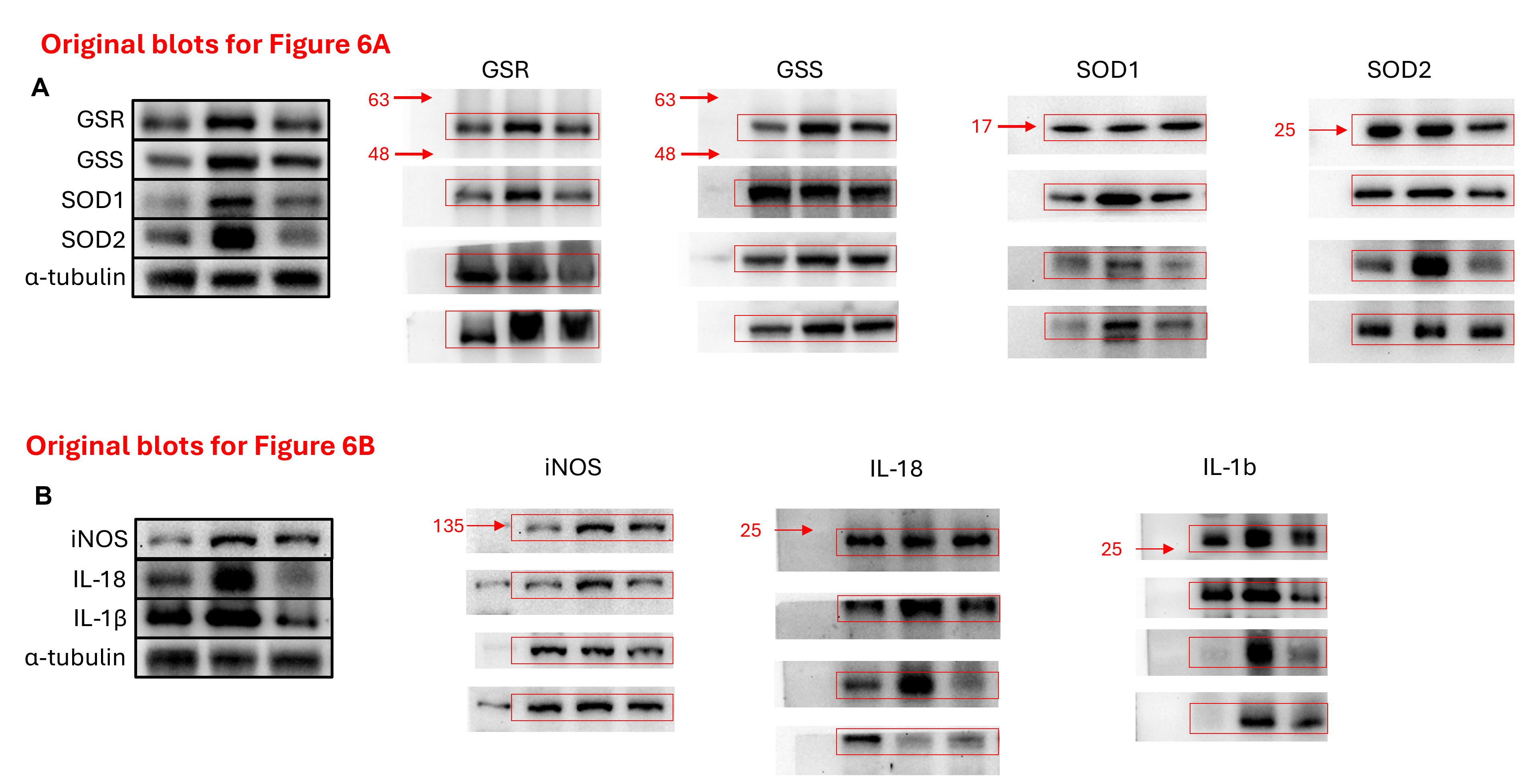

Supplement: S2 Fig — (TIF) [file pntd.0013825.s003.tif]
